# Localization in Diseases of the Brain

**Published:** 1879-04

**Authors:** 


*Lecture on Localization in Diseases of the Brain.* Delivered at the Facultie De Medécine Paris, 1875. By J. M. Charcot, Professor in the Faculty of Medicine of Paris. Translated by Edward T. Fowler, M. D., New York. William Wood & Co. 27 Great Jones street, New York.

The author prefaces his Lectures by "I have selected information furnished by normal Anatomy, experimental physiology and clinical observation, illustrated by methodical examination of organic lesions. I have always given precedence however, to the last mentioned order of testimony. Convinced that, although normal Anatomy and experimental Physiology may serve to indicate the true direction; still Clinical and Pathological research are necessary (in case of the human subject) to the final judgment as to the *furnishing proof.*"

The work comprises Twelve Lectures, with illustrative cuts of brain organs and brain diseases, and cannot fail to interest and instruct the careful reader. It is a vigorous attempt to illustrate and explain the obscure, undetermined and unlocated diseases of the brain.

